# The role of autophagy in pancreatic diseases

**DOI:** 10.3389/fphar.2024.1444657

**Published:** 2024-09-30

**Authors:** Wen-Gang Zhang, Qing-Zhen Wu, Bo-Zong Shao

**Affiliations:** Department of Gastroenterology, General Hospital of the Chinese People’s Liberation Army, Beijing, China

**Keywords:** pancreatic disease, autophagy, pancreatitis, pancreatic cancer, inflammation

## Abstract

Pancreatic diseases such as pancreatitis and pancreatic cancer represent significant health challenges characterized by high mortality rates and limited survival durations. Autophagy, a crucial cellular catabolic process, has emerged as a focal point in understanding various pathological conditions, spanning inflammation-related disorders to malignant neoplasms. This comprehensive review aims to elucidate the biological intricacies of autophagy and its pivotal roles within two extensively researched pancreatic diseases, namely pancreatitis and pancreatic cancer, drawing upon recent scholarly contributions. The discussion will delve into the nuanced mechanisms underlying autophagy’s involvement in these diseases, shedding light on its potential as a therapeutic target. Furthermore, the review will explore cutting-edge therapeutic interventions leveraging autophagy regulation for managing pancreatitis and pancreatic cancer. Through this analysis, we endeavor to offer novel insights into the pathophysiology of pancreatic disorders and contribute to the development of innovative therapeutic modalities in this challenging clinical domain.

## Introduction

The pancreas plays a pivotal role as a secretory organ within the human body, comprising two distinct components: the exocrine and endocrine divisions (Czako et al., 2009; Das et al., 2014; Zhou and Melton, 2018). The exocrine segment encompasses acinar and ductal structures responsible for secreting pancreatic juice, a fluid containing vital enzymes such as trypsinogen, lipase, and amylase (Czako et al., 2009; Das et al., 2014; Zhou and Melton, 2018). This pancreatic fluid, essential for the digestion of proteins, fats, and sugars, is conveyed into the duodenum via the pancreatic duct network (Czako et al., 2009; Das et al., 2014; Zhou and Melton, 2018).

Comprising primarily pancreatic acinar and ductal cells (Wang et al., 2019; Huang et al., 2021; Grimont et al., 2022), any impairment or malfunction in these cellular components may precipitate pancreatic disorders, including pancreatitis and pancreatic cancer. However, due to the intricate nature of pancreatic pathology, the development of optimal therapeutic approaches remains imperative to combat such diseases effectively.

Autophagy, recognized as a critical metabolic process (Xu and Wan, 2023), facilitates the degradation and recycling of long-lived proteins, misfolded proteins, and redundant organelles during stress conditions, predominantly reliant on lysosomal activity (Debnath et al., 2023; Jain et al., 2023; Samare-Najaf et al., 2023). Initially documented in the mid-1950s, autophagy has since been implicated in the pathogenesis and progression of diverse disease states (Kumaresan et al., 2022; Yim et al., 2022; de Abreu et al., 2023). Notably, in the context of pancreatic disorders, extensive research has elucidated autophagy’s regulatory roles in the initiation and progression of pancreatitis and pancreatic cancer (Chen et al., 2024b; Cui et al., 2024; Liu et al., 2024b; Zhang et al., 2024). Contemporary investigations suggest that targeting autophagy holds promise as a therapeutic avenue for managing pancreatic disorders (Liu et al., 2023; Ashrafizadeh et al., 2024; Elango et al., 2024).

Building upon this foundation, this paper aims to comprehensively review and discuss the latest literature pertaining to the role of autophagy in pancreatitis and pancreatic cancer. Through this exploration, we endeavor to offer novel insights into the treatment of pancreatic disorders by leveraging the potential of autophagy modulation.

## Biological features of autophagy

Autophagy represents a fundamental catabolic cellular process responsible for the degradation of protein aggregates and damaged organelles into metabolic components via lysosomal recycling, thereby maintaining cellular homeostasis and vitality (Miller and Thorburn, 2021; Liu et al., 2024a). This process is ubiquitously present in virtually all cell types and evolutionarily conserved from yeast to mammals (Feng et al., 2024; Proikas-Cezanne and Thumm, 2024; Riaz et al., 2024). Autophagy manifests in three primary forms based on cargo delivery and physiological function: macroautophagy, microautophagy, and chaperone-mediated autophagy (Kuchitsu and Taguchi, 2023; Yamamoto and Matsui, 2024). Macroautophagy, the most extensively studied form, entails the sequestration of degradable materials within double-membraned autophagosomes, subsequently fusing with lysosomes for degradation (Al-Kuraishy et al., 2024; Lewerissa et al., 2024). Microautophagy, in contrast, involves the non-selective engulfment of cytoplasmic materials through lysosomal/vacuolar membrane invagination (Alvarez-Guerra et al., 2024; Sakai and Oku, 2024). Chaperone-mediated autophagy is a selective process reliant on chaperone-mediated recognition of substrate proteins with specific motifs and lysosomal chaperones (Dong et al., 2021; Wang et al., 2023c). Since macroautophagy is, so far, the best-studied autophagy process, this review will mainly discuss the roles and mechanisms of macroautophagy in pancreatic disorders including pancreatitis and pancreatic cancer (hereafter referred to as “autophagy”).

**FIGURE 1:**
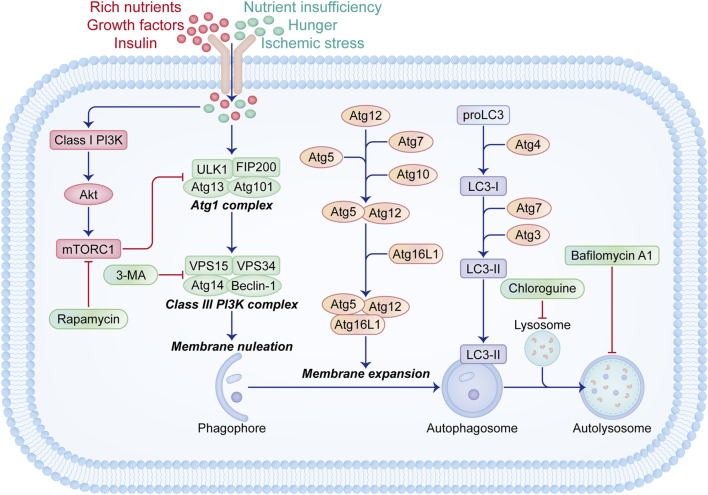
Schematic illustration of signaling pathways related to the initiation and regulation of autophagy. Autophagy is activated during nutrient deprivation, starvation, or ischemic stress conditions. The initiation of autophagy involves various groups of Atg proteins. The Atg1 complex, consisting of ULK1, FIP200, Atg13, and Atg101, initiates the assembly of the Class III PI3K complex containing Beclin-1, Atg14, VSP15, and VSP34, which promotes membrane nucleation and phagophore formation. Subsequent membrane expansion and fusion mediated by Atg5-Atg12-Atg16L1 and LC3-II lead to autophagosome formation, followed by fusion with lysosomes to form functional autophagy units. Under conditions of ample nutrients, growth factors, and insulin, the Class I PI3K-Akt-mTORC1 signaling pathway is activated, inhibiting the Atg1 complex formation. Rapamycin acts as an autophagy inducer by inhibiting mTORC1, while 3-MA, bafilomycin A1, and chloroquine block autophagy through distinct mechanisms.

The induction of autophagy comprises two essential steps, as elucidated in our prior reviews (Figure 1) (Wang et al., 2018; Shao et al., 2021; Shao et al., 2022a). Initially, substrate materials like aggregated proteins are enveloped by cup-shaped photophores with lipid bilayer membranes, leading to their sequestration within double-membrane autophagosomes. Subsequently, autophagosomes shed “coat proteins (LC3-II)” from their surfaces and merge with lysosomes to form functional autolysosomes. Over 30 autophagy-related genes (Atgs) are implicated in these processes. Furthermore, two pivotal signaling pathways regulate autophagy: the Class I PI3K-mammalian target of rapamycin (mTOR) pathway acts as an inhibitory regulator via mTOR complex 1 (mTORC1) stimulation (Borsa et al., 2024; Huang et al., 2024), while the Class III PI3K-Beclin-1 complex mediates an inductive pathway (Liu et al., 2021; Yan et al., 2023).

In recent years, extensive research has explored the role of autophagy in various disease contexts, including cardiovascular disorders like myocardial infarction (Grazide et al., 2024; Li et al., 2024b) and atherosclerosis (Shao et al., 2023; Xu et al., 2024b), neurodegenerative conditions such as multiple sclerosis (Ke et al., 2017; Shao et al., 2017), metabolic diseases like diabetes (Yang et al., 2024; Yasasilka and Lee, 2024) and obesity (Behrooz et al., 2024; Li et al., 2024a), inflammatory and immune-related disorders such as pancreatitis (Wang et al., 2023a; Fu et al., 2024), inflammatory bowel disease (Shao et al., 2019; Shao et al., 2022b), and arthritis (Wang et al., 2024), as well as malignant tumors (Chen et al., 2024a; Mathur et al., 2024; Yu et al., 2024). While autophagy’s anti-inflammatory and immune-suppressive effects are well-documented, it has also been associated with cellular death termed “autophagic cellular death” in specific circumstances (Lin et al., 2024; Sakil et al., 2024; Xu et al., 2024a). These complexities highlight the intricate roles of autophagy in disease pathogenesis. Subsequently, this paper will delve into the detailed discussion of autophagy’s involvement in pancreatic disorders, including pancreatitis and pancreatic cancer.

## Autophagy in pancreatic diseases

### Autophagy in pancreatitis

#### The role of autophagy in pancreatitis

Pancreatitis, characterized by trypsin-induced self-digestion of the pancreas, manifests with edema, congestion, bleeding, or necrosis (Liu et al., 2019; Pesei et al., 2022). Clinical symptoms include abdominal pain, bloating, nausea, vomiting, and fever (Nguyen et al., 2023). This disease encompasses acute and chronic forms, with acute pancreatitis being a significant cause of gastrointestinal-related hospital admissions. While most cases of acute pancreatitis are mild to moderate, up to 30% of severe cases with persistent multi-organ dysfunction can be fatal (Lilja et al., 2008; Gukovskaya et al., 2017; Yoshimi et al., 2022). Chronic pancreatitis may arise from repeated subclinical or clinically evident episodes of acute pancreatitis, or develop independently (Lilja et al., 2008; Gukovskaya et al., 2017; Yoshimi et al., 2022). Contributing factors include genetic predisposition, gallstones, and alcohol misuse (Gukovskaya et al., 2017). The prevailing understanding posits acinar cell injury as the initial trigger, leading to parenchymal necrosis and inflammation, central to the disease’s pathology (Yu et al., 2023; Zaman and Gorelick, 2024). In addition, certain vacuole-associated proteins, including LC3 protein, lysosomal-associated membrane protein (LAMP)-1, and LAMP-2 in autophagic vacuoles, have been revealed to contribute greatly to the modulation of pancreatitis (Gukovsky et al., 2012; Mareninova et al., 2020). Besides, cholecystokinin (CCK), a major gastrointestinal hormone that plays an important role in stimulation of pancreatic secretion, has been revealed in high plasma level in patients with acute pancreatitis (Otsuki, 2000). Treatment of CCK-8 and CCK-1 receptor antagonist significantly alleviate pancreatitis through attenuating acinar necrosis in pancreas (Jia et al., 2015; Grupp et al., 2020). Emerging evidence suggests that systemic complications in acute pancreatitis stem from dysregulated immune system activation (Kessler et al., 2020).

We searched in PubMed and other digital sources with the keywords of “autophagy” and “pancreatitis” and more than 2400 studies have been retrieved. According to those previous findings, impaired autophagy plays a pivotal role in the pathogenesis and progression of pancreatitis. Studies have shown a heightened basal level of autophagy in mouse exocrine pancreas (Mizushima et al., 2004). In pancreatitis patients, increased levels of LC3-II and p62 in acinar cells alongside decreased LAMPs levels, indicative of impaired lysosomal function, have been observed (Mareninova et al., 2015; Mareninova et al., 2020; Yang et al., 2022). Autophagic vacuole accumulation, notably large autolysosomes, during pancreatitis points to inefficient lysosomal degradation (Mareninova et al., 2009; Gukovskaya and Gukovsky, 2012). Genetic modifications of core autophagy-related proteins like Atg5, Atg7, or LAMP2 in the pancreas lead to severe acinar cell degeneration, exocrine pancreas atrophy, fibrosis, inflammation, and cellular damage (Antonucci et al., 2015; Mareninova et al., 2015), highlighting autophagy impairment’s role in exacerbating pancreatitis severity. As recently reviewed by Tsomidis et al. (Tsomidis et al., 2024), autophagy plays an important role in pancreatitis through the connection of many mechanisms including endoplasmic reticulum (ER) stress, immunity, cell death process, and inflammation.

It is worth noting that the overload of calcium ion, an important secondary messenger, contributes to the pathogenesis and progression of pancreatitis through the regulation of impaired autophagy (Booth et al., 2011; Ono et al., 2023). Calcium ions have been revealed to regulate pancreatic secretion, acidic stores in pancreatic acinar cells, and lysosomal pH value, thus maintaining pancreatic homeostasis (Maleth and Hegyi, 2014; Petersen et al., 2021). It was recently revealed that the disturbance of calcium iron overload in pancreatic acinar cells via microRNA-26a significantly suppressed the severity of acute pancreatitis (Du et al., 2022). Such effect was mediated by the inhibition of impaired autophagy as well as inflammatory reaction and endoplasmic reticulum (ER) stress (Du et al., 2022).

Several signaling pathways regulate autophagy in pancreatitis. The STING/TBK1/IRF3 pathway modulates autophagy levels in cerulein-treated acinar cells (Song et al., 2021). The microRNA-30b-5p/CAMKII pathway contributes to impaired autophagy promotion in pancreatitis facilitated by Atg7 (Ji et al., 2022). The AMPK/mTOR pathway, a classic autophagy regulator, induces autophagy in pancreatitis (Ji et al., 2016). Furthermore, autophagy interacts with ferroptosis and inflammation mechanisms, exacerbating acute pancreatitis via impaired autophagy-induced ferroptosis (Yang et al., 2023) and strengthening the intestinal mucosal barrier during severe acute pancreatitis by attenuating oxidative stress (Huang et al., 2018). Crosstalk between autophagy and NF-κB-mediated inflammation is also noted in pancreatitis (Yang et al., 2012).

#### Pharmacological application of autophagy in pancreatitis

Numerous agents have demonstrated efficacy in mitigating pancreatitis by leveraging autophagy regulation (shown in Table 1). Rapamycin, a well-studied autophagy inducer, has shown considerable promise in ameliorating the pathogenesis and progression of pancreatitis. Mei et al. (Mei et al., 2020) reported rapamycin’s ability to alleviate hypertriglyceridemia-related acute pancreatitis by restoring autophagy flux and inhibiting endoplasmic reticulum stress, achieved through mTORC1/S6K1 signaling pathway inhibition. Müller et al. (Muller et al., 2012) demonstrated rapamycin’s potential to mitigate severe acute pancreatitis severity, possibly by early suppression of helper T cells. Additionally, rapamycin administration has been shown to mitigate oxidative stress and prevent apoptotic cell death in experimental chronic pancreatitis (Ozturk et al., 2015).

**TABLE 1:** Potential pharmacological mechanisms of autophagy regulators in pancreatitis treatment.

Autophagy regulators		Potential pharmacological mechanism	Clinical trial	References
Type	Name			
Classic autophagy inducer	Rapamycin	Inhibiting mTORC1/S6K1 signaling pathway; early suppression of helper T cells; alleviating oxidative stress	NA	Mei et al. (2020), Muller et al. (2012), Ozturk et al. (2015)
Natural compounds	Xanthohumol	Inhibiting AKT/mTOR signaling pathway	NA	Huangfu et al. (2023)
	Picroside II	Inducing NF-κB-dependent autophagy	NA	Piao et al. (2017)
Other agents	α7nAChR agonist	Activating cholinergic anti-inflammatory pathway; enhancing TFEB-regulated autophagy	NA	Li et al. (2020)
	β1 syntrophin	Facilitating autophagy initiation	NA	Ye et al. (2019)
	MicroRNAs (microRNA-20b-5p, microRNA-148a, microRNA-551b-5p, and microRNA-141)	Targeting AKT3 signaling pathway; regulating IL-6/STAT3 signaling pathway; Regulating HMGB1/beclin-1 signaling pathway	Wei et al. (2024)	Zhu et al. (2016), Tang et al. (2021), Wei et al. (2024)

Several natural compounds have also exhibited efficacy in pancreatitis alleviation. Xanthohumol, derived from hops, attenuates severe acute pancreatitis by suppressing oxidative stress and restoring impaired autophagy via AKT/mTOR signaling pathway inhibition (Huangfu et al., 2023). Picroside II, from Picrorhiza scrophulariiflora Pennell, has shown efficacy in severe acute pancreatitis alleviation by enhancing antioxidant and anti-inflammatory activities through NF-κB-dependent autophagy modulation (Piao et al., 2017).

Other agents have been identified for their pancreatitis alleviation potential. Activation of α7 nicotinic acetylcholine receptor (α7nAChR), integral to the cholinergic anti-inflammatory pathway, protects against acute pancreatitis by enhancing transcription factor EB (TFEB)-regulated autophagy (Li et al., 2020). Additionally, β1 syntrophin, a critical regulator of actin cytoskeleton, shields against cerulein-induced acute pancreatitis by facilitating autophagy initiation in pancreatic acinar cells (Ye et al., 2019). Several microRNAs, including microRNA-20b-5p, microRNA-148a, microRNA-551b-5p, and microRNA-141, have been implicated in pancreatitis regulation through autophagy modulation (Zhu et al., 2016; Tang et al., 2021; Wei et al., 2024).

### Autophagy in pancreatic cancer

#### The role of autophagy in pancreatic cancer

Pancreatic cancer stands out as one of the deadliest malignancies primarily due to delayed diagnosis, high metastatic potential, and aggressive local spread (Gorgulu et al., 2020). Despite the availability of various treatment modalities such as surgical resection, radiation therapy, immunotherapy, and chemotherapy, the median survival for pancreatic cancer patients remains a mere 6–9 months with a dismal 5-year survival rate of 9% (Rawla et al., 2019). Among pancreatic cancer subtypes, pancreatic ductal adenocarcinoma (PDAC) prevails as the most common, constituting approximately 3% of all cancers (Jiang et al., 2024). Noteworthy epidemiological risk factors for PDAC encompass smoking, excessive alcohol consumption, obesity, sedentary lifestyle, inadequate consumption of fruits and vegetables, and high-fat intake (Asahina et al., 2020; Li et al., 2021). These factors serve as significant extrinsic contributors to tumorigenesis, while gene mutations, particularly in *Kras* (*Kras* protooncogene, GTPase), *Tp53* (tumor protein p53), *Smad4* (Smad family member 4), and *Brca2* (Brca2 DNA repair associated), represent intrinsic triggers for pancreatic tumorigenesis (Li et al., 2021; de Jesus et al., 2023; Gurreri et al., 2023). Besides, several vacuole-associated proteins, such as LC3B and vacuole membrane protein 1 (VMP1), play an important role in pancreatic cancer (Lima et al., 2023; Renna et al., 2023). The interplay of intrinsic and extrinsic factors disrupts cellular homeostasis, profoundly influencing tumorigenesis in both cell-autonomous and non-cell-autonomous manners.

We searched in PubMed and other digital sources with the keywords of “autophagy” and “pancreatic cancer” and more than 1300 studies have been retrieved. According to those previous findings, autophagy, a pivotal process in maintaining cellular homeostasis, plays a dual and intricate role in the pathogenesis and progression of pancreatic cancer, influenced by factors such as tumor stage, duration of exposure, and mode of autophagy modulation. Studies have shown significant upregulation of key autophagy genes like beclin-1 and LC3 in both mRNA and protein levels in tumor tissues compared to normal tissue (Cui et al., 2019). Elevated expression of these genes correlates with advanced TNM stages, lymphatic metastasis, and poor patient outcomes (Cui et al., 2019). PDAC cells exhibit heightened autophagy and lysosomal activity by uncoupling microphthalmia/transcription factor E (MiT/TFE) family transcription factors from mTOR signaling (Settembre et al., 2011; Ko et al., 2013; Perera et al., 2015). Additionally, PDAC cells effectively maintain basal autophagy levels by dephosphorylating ULK1 via PTPA/protein phosphatase 2 phosphatase activator (PP2A) (Wong et al., 2015).

Various signaling pathways related to cellular proliferation, apoptosis, and metabolism are implicated in autophagy’s impact on pancreatic cancer. For instance, concurrent inhibition of MAPK/ERK and IGF1R pathways enhances sensitivity to autophagy inhibitors in PDAC (Stalnecker et al., 2022a). Similarly, targeting IGF1R and ERK together enhances the efficacy of autophagy inhibitors (Stalnecker et al., 2022b). The microRNA454-FAM83A-TSPAN1 axis has been identified as a crucial regulator of pancreatic cancer cell proliferation through autophagy modulation (Zhou et al., 2021). Moreover, the intricate interplay between autophagy and inflammation further complicates the understanding of pancreatic cancer pathogenesis. Conditional knockout of Atg7 in pancreatic epithelial cells leads to acinar cell degeneration and inflammation, highlighting the multifaceted role of autophagy in pancreatic cancer progression (Antonucci et al., 2015; Zhou et al., 2017).

For the role of autophagy in tumor resistance to antineoplastic agents, it has been revealed that autophagy is highly involved in regulation of the long noncoding RNA PVT1-mediated gemcitabine resistance of pancreatic cancer via activating Wnt/β-catenin signaling pathway (Zhou et al., 2020). In addition, cancer-associated fibroblast autophagy was shown to contribute to pancreatic cancer resistance to immune surveillance and caner immunotherapy through modulating CD274/PDL1 levels (Zhang et al., 2024). Those findings indicate the close connection between autophagy and tumor resistance to antineoplastic therapies.

#### Pharmacological application of autophagy in pancreatic cancer

Numerous agents have demonstrated effectiveness in treating pancreatic cancer by modulating autophagy (shown in Table 2). Classic autophagy inhibitors, such as bafilomycin A1, have shown promise in lowering pHi, increasing thermosensitivity, and enhancing heat-induced growth delay in pancreatic cancer cells through vacuolar type H^+^ATPase inhibition and apoptosis induction (Ohta et al., 1998; Hayashi et al., 2006). Chloroquine (CQ) and hydroxychloroquine (HCQ), widely used autophagy inhibitors, have also been effective in mitigating pancreatic cancer growth by triggering autophagy-mediated inflammation, impacting cell survival via signaling pathways like CXCL12/CXCR4, ERK/STAT3, and RAGE/STATs (Balic et al., 2014; Chen et al., 2021a; McCubrey et al., 2023). Several clinical trials are available for CQ and/or HCQ with or without the combination of other classic chemotherapeutics in the treatment of pancreatic cancer (Samaras et al., 2017; Zeh et al., 2020).

**TABLE 2:** Potential pharmacological mechanisms of autophagy regulators in pancreatic cancer treatment.

Autophagy regulators		Potential pharmacological mechanisms	Clinical trial	References
Type	Name			
Classic autophagy inhibitors	Bafilomycine A1	Inhibiting vacuolar type H^+^ATPase and inducing apoptosis	NA	Ohta et al. (1998), Hayashi et al. (2006)
	CQ and HCQ	Regulating CXCL12/CXCR4, ERK/STAT3, and RAGE/STATs signaling pathways	Samaras et al. (2017), Zeh et al. (2020)	Balic et al. (2014), Chen et al. (2021a), McCubrey et al. (2023), Samaras et al. (2017), Zeh et al. (2020)
Natural compounds	Danthron	Sensitizing the chemotherapeutic effect of doxorubicin	NA	Chen et al. (2019)
	Qingyihuaji Formula	Inhibiting MAPK/ERK and PI3K/Akt/mTOR signaling pathways	NA	Qian et al. (2023)
	Umbelliprenin	Regulating autophagy-related BxPC3 apoptosis	NA	Wang et al. (2023b)
	Selaginellin B	Inducing JAK2 signaling pathway	NA	Chu et al. (2020)
Oother small molecular compounds	Metformin	Inducing AMPK-related autophagy process	Kordes et al. (2015), Reni et al. (2016)	Chen et al. (2021b), Kordes et al. (2015), Reni et al. (2016)
	Fructose	Inducing mTORC1 signaling pathway	NA	Cui et al. (2023)
	GSK343	Downregulating the AKT/mTOR signaling pathway	NA	Xu et al. (2019)

Natural compounds have also emerged as effective tools in pancreatic cancer alleviation through autophagy modulation. Danthron, from Rheum palmatum L., sensitizes pancreatic cancer cells to doxorubicin chemotherapy by suppressing autophagy (Chen et al., 2019). Qingyihuaji Formula, a traditional Chinese medicine, alleviates pancreatic cancer by modulating autophagy via MAPK/ERK and PI3K/Akt/mTOR pathways (Qian et al., 2023). Umbelliprenin from Artemisia absinthium L. inhibits tumor growth and induces apoptosis in pancreatic cancer cells through autophagy regulation (Wang et al., 2023b). Selaginellin B from Selaginella tamariscina targets Janus kinase 2 (JAK2) to treat pancreatic cancer via autophagy modulation (Chu et al., 2020).

Furthermore, small molecular compounds have exhibited anti-tumor effects in pancreatic cancer. Metformin combined with pitavastatin shows promise as a chemotherapeutic agent by inducing AMPK-related autophagy (Chen et al., 2021b). Several clinical trials are available to demonstrate the therapeutic effect of metformin on pancreatic cancer (Kordes et al., 2015; Reni et al., 2016). Fructose-induced mTORC1 activation promotes pancreatic cancer progression by inhibiting autophagy, highlighting potential strategies for targeting metabolism in PDAC treatment (Cui et al., 2023). GSK343, an EZH2 inhibitor, demonstrates anti-tumor effects by inducing autophagy and downregulating AKT/mTOR signaling in pancreatic cancer cells (Xu et al., 2019).

## Conclusion

Recent research has shed light on autophagy’s involvement in the pathogenesis and progression of two extensively studied pancreatic disorders, namely pancreatitis and pancreatic cancer. Furthermore, we have witnessed the effectiveness of several autophagy-related therapies in mitigating these conditions. As discussed above, various agents leveraging autophagy have shown efficacy in treating both pancreatitis and pancreatic cancer. These agents encompass natural compounds as well as classic autophagy modulators, whether inducers or inhibitors. However, despite extensive investigations into the mechanisms of pancreatic diseases, the precise molecular pathways remain elusive, hindering the development of innovative treatments. Moreover, given the intricate impact of autophagy on pancreatitis and pancreatic cancer, further research is imperative to translate autophagy-based therapeutic strategies into clinical practice effectively.
